# Pacemaker lead malpositioning led to subsequent ischemic strokes despite antiplatelet and anticoagulation therapy

**DOI:** 10.1186/1749-8090-9-54

**Published:** 2014-03-20

**Authors:** Claus Rath, Martin Andreas, Caesar Khazen, Dominik Wiedemann, Andreas Habertheuer, Alfred Kocher

**Affiliations:** 1 Division of Cardiac Surgery, Vienna General Hospital, Medical University of Vienna, Waehringer Guertel 18-20, Vienna A-1090, Austria

**Keywords:** Pacemaker, Lead malpositioning, Left ventricle, ECG, Chest-X-ray, Echocardiography

## Abstract

Pacemaker lead malpositioning may lead to severe clinical adverse events. Rarely, cases of inadvertent placement of a lead into the left ventricle are reported in the literature. We herein report a case of pacemaker lead malpositioning into the left ventricle via a persistent foramen ovale in a male caucasian patient. After this procedural adverse event, the patient suffered from two ischemic strokes despite antiplatelet and anticoagulation therapy.

## Background

While unidentified malpositioning of a pacemaker lead is already a rare complication at all, only a few cases of inadvertently placement of a lead into the left ventricle are reported in the literature. Neither incidence, nor clinical history of this complication are well known.

Malpositioning of a lead may cause structural damage or trigger thromboembolic events. Although lead repositioning is the treatment of choice, there are no data about procedurals risks, neither are there distinct recommendations to improve safety of these high-risk interventions.

## Case presentation

A 75-year-old, male, caucasian patient was admitted to the neurology department of a peripheral hospital due to an ischemic stroke in the supply area of the left posterior cerebral artery.

Patient’s history revealed type 2 diabetes mellitus and coronary heart disease with previous NSTEMI and subsequent stenting. Furthermore, a prior thrombosis in the popliteal vein was reported. Current medication was 50 mg acetylsalicylic acid, an angiotensin-converting-enzyme inhibitor, hydrochlorothiazide and metformin. The patient underwent implantation of a permanent dual chamber pacemaker (Medtronic Ensura EN1DR01 MR) one month before admission due to a Sick-Sinus-Syndrome.

**Figure 1:**
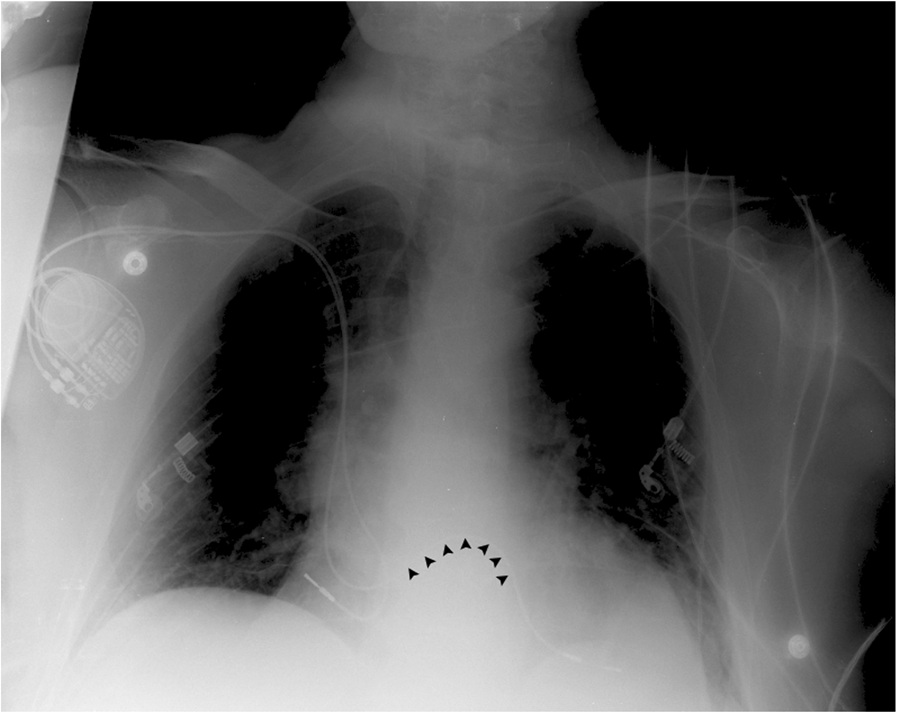
**Chest X-ray.** The ventricular pacemaker lead follows an unusual course (black arrows) due to its placement via a PFO into the left ventricle.

**Figure 2:**
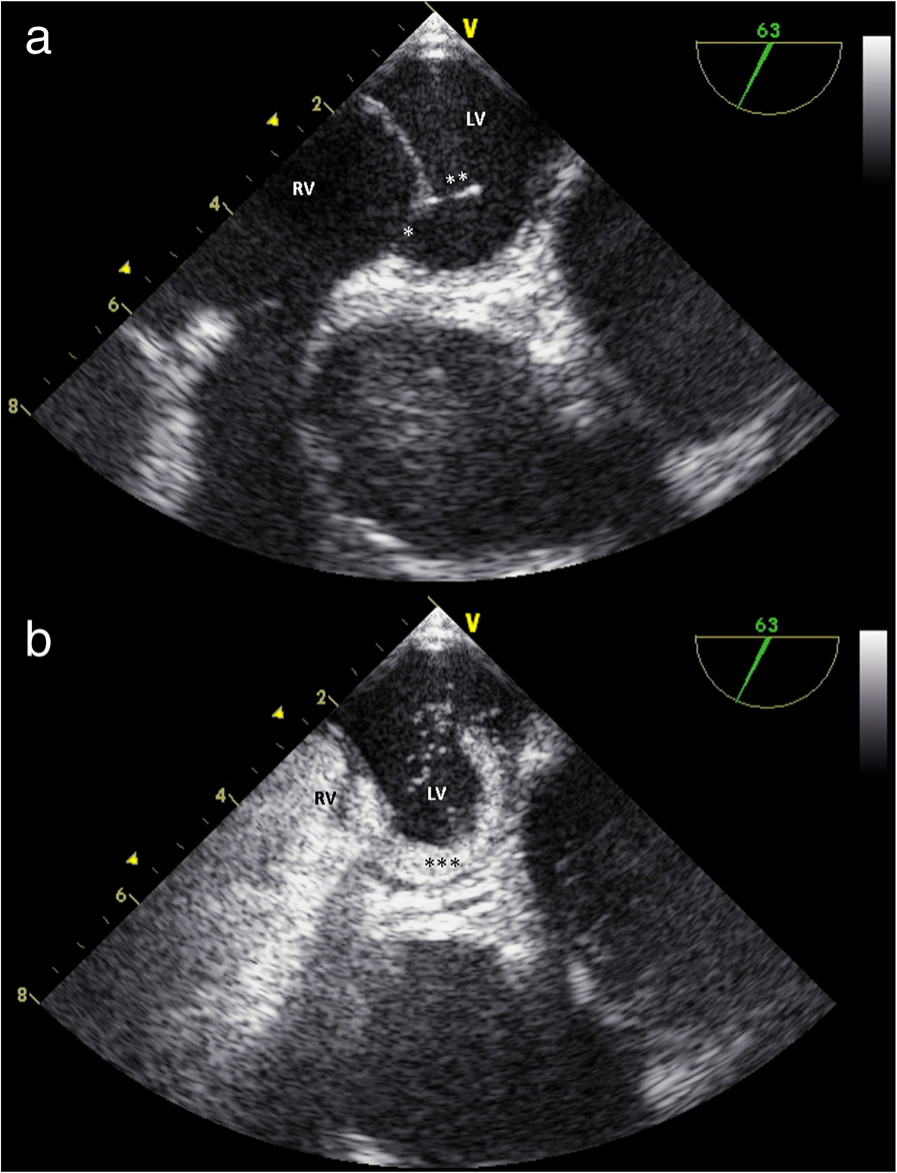
**a and b: Transoesophageal echocardiography revealed a PFO.** LV left ventricle; RV right ventricle; * PFO, 8 mm; ** pacemaker lead; *** contrast agent.

The patient was primarily loaded with 325 mg acetylsalicylic acid and low-molecular-weight heparin. The oral diabetes medication was stopped. Since the stroke occurred under the therapy with acetylsalicylic acid, it was replaced by clopidogrel (75 mg per day). Additionally, low-molecular-weight heparin (LMWH) in therapeutic dose was prescribed. The pacemaker control showed inconspicuous parameters. The pacing thresholds, the impedance of the electrodes and the sensing were normal. A self-limiting episode supraventricular tachycardia was recorded, another episode occurred during the control. Diagnostic assessment of cardiac risk factors was done. A chest X-ray gave a first hint for a problem with the ventricular lead, since it was deflected and followed an unusual course (Figure 1). Transesophageal echocardiography revealed a patent foramen ovale (PFO) with a diameter of 8 mm and malpositioning of the ventricular pacemaker lead. This lead was unintentionally malpositioned via the PFO into the left ventricle. The atrial lead was correctly placed at the bottom of the right atrium (Figure 2). Echocardiography didn’t reveal any thrombotic material on the ventricular lead.

While still being hospitalised, twenty-two days after the admission and eighteen days after detection of the lead malpositioning, the patient suffered of another ischemic stroke - located in the supply area of the right posterior cerebral artery and in the left cerebellar region. After stabilization, the patient was transferred to our department for re-positioning of the ventricular lead and closure of the PFO.

**Figure 3:**
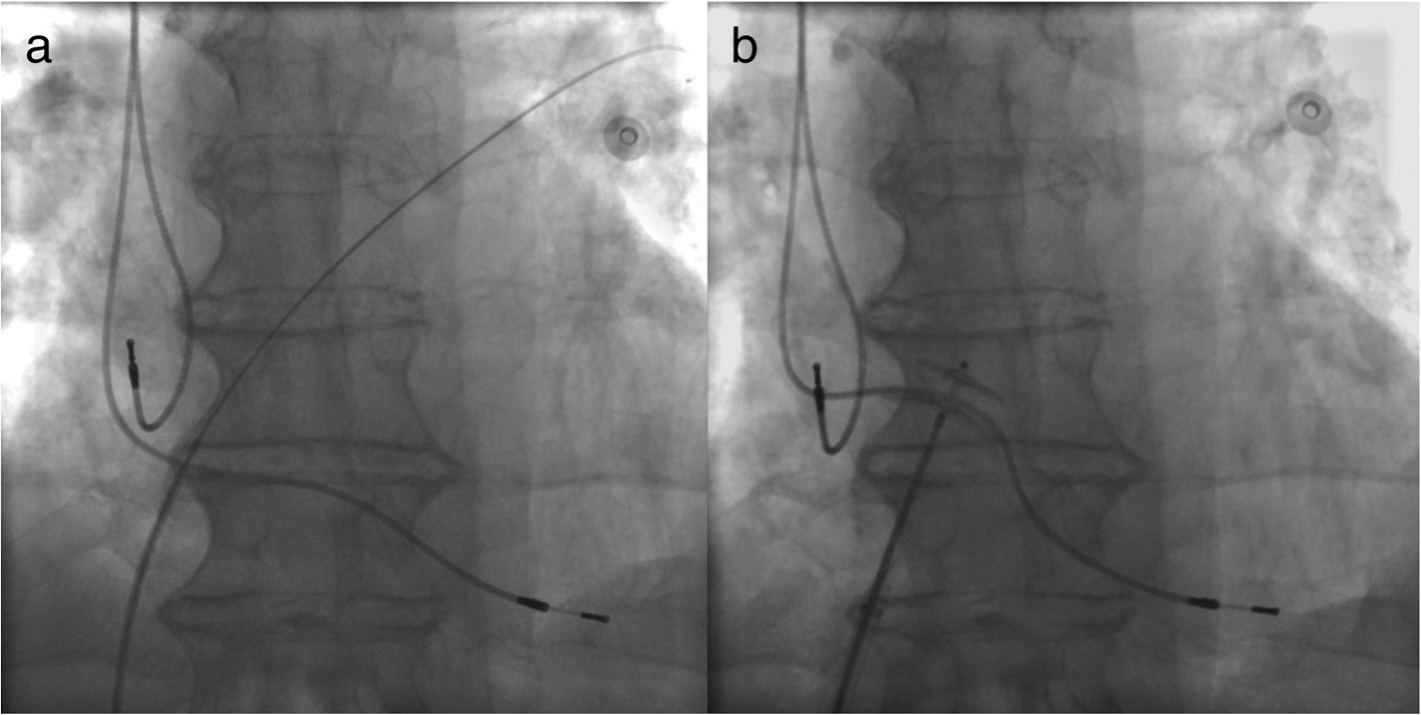
**a and b: Chest fluoroscopy.** A few days after the re-positioning of the ventricular lead, the PFO was closed with a 25 mm Amplatzer® Septal Occluder.

In a first session, in general anaesthesia, using a percutaneous approach, the lead was retracted and repositioned into the right ventricle. A few days later, the PFO was closed with a 25 mm Amplatzer® Septal Occluder in a second session (Figure 3). After an uneventful postoperative course, the patient was transferred to a peripheral hospital for neurorehabilitation.

## Conclusion

Though pacemaker implantation is a common procedure, the complication rates associated with pacemaker implantation vary between 1.7% [1] and 12.4% [2] according to recent studies. Up-to-date data regarding pacing-lead induced thrombotic and thromboembolic events in general population are not available. Data from the 1970s and 1980s suggest an event rate from 0.6% to 3.5% [3-7]. In patients with intracardiac shunts, event rates from 0.5% to 0.7% per year were identified. In patients with intracardiac shunts and transvenous pacing, risk for systemic thromboemboli is twice as high. For this group of patients, neither for aspirin nor for warfarin a positive effect could be demonstrated [8].

To our best knowledge, the presented case is the first one in the literature, where a misplaced pacing lead into the left ventricle led to ischemic strokes in a patient with both, prophylactic anticoagulation and antiplatelet therapy. At the time the patient suffered of the second stroke, his coagulation parameters were within a normal range. The creatinine levels remained throughout the stay between 0,97 mg/dl and 1.16 mg/dl. However, we cannot completely exclude the possibility of undertreatment with LMWH. Furthermore, paroxysmal atrial fibrillation might also be a reason for the recurrent stroke, although AF had not been reported in the patient’s medical history and the ECG did not show any signs of AF.

In permanent pacemaker implantation, some manoeuvres may increase the secure lead placement in the right ventricle. First, the lead should be introduced with a loop through the tricuspid valve and not directly with the tip to avoid placement in the coronary sinus. Further, the occurrence of ventricular extrasystoles is a good indicator for being in the ventricle. Then, the lead could be advanced with the tip into the pulmonary outflow tract or pulmonary artery to confirm the correct position. Thereafter, the lead is pulled back and advanced into the apex of the right ventricle. If the lead measurements are not in the recommended range, lead placement should be repeated.

Two view chest-X-ray and an ECG should be performed routinely after pacemaker implantation.

Even if the electrode seems to be in the correct position on the frontal radiograph, the lateral view may reveal a malposition of an electrode.

If the ventricular lead was placed correctly into the right ventricle, one would expect a left bundle branch block pattern when taking a 12-lead ECG (electrocardiogram). A right bundle branch block pattern should raise suspicion about the placement of the lead. This should be controlled after every implantation, which may not always be the case.

**Figure 4:**
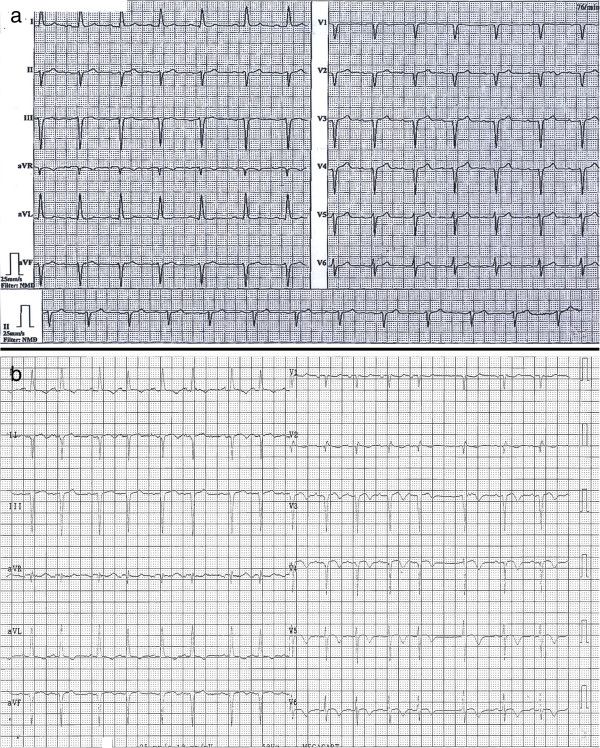
a and b: ECGs before and after repositioning of the ventricular pacemaker lead did not show any signs for pacemaker lead malpositioning.

In our patient, the ECG did not show any alarming abnormalities after the initial pacemaker implantation. It was classified as Sinus rhythm at 76 bpm with left axis deviation. PQ-interval, QRS complex, ST-segment and QT-interval were within a normal range. A left anterior fascicular block was diagnosed. Myocardial ischemia of the posterior wall was known from the patient’s medical history (Figure 4a). After repositioning of the lead, the ECG was interpreted as Sinus rhythm at 95 bpm with left axis deviation, left anterior hemi block and repolarisation disorder (Figure 4b).

A few cases have been reported, where patients didn’t suffer of any thromboembolic events despite a pacing lead placed into the left ventricle via a PFO [9-11].

However, the thrombogenic potential of pacemaker leads may be underestimated in clinical routine, especially in patients with intracardial shunting and pre-existing myocardial damage.

This case underlines the urgent surgical revision of misplaced pacemaker leads. It highlights the importance of echocardiography for post-surgical control in difficult patients or in patients with signs of lead malpositioning.

## Consent

Written informed consent was obtained from the patient for publication of this Case report and any accompanying images. A copy of the written consent is available for review by the Editor-in-Chief of this journal.

## Abbreviations

AF: Atrial fibrillation; BPM: Beats per minute; ECG: Electrocardiogram; LMWH: Low molecular weight heparin; PFO: Patent foramen ovale.

## Competing interests

The authors declare that they have no competing interests.

## Authors’ contributions

CR: prepared the manuscript, drafted the manuscript. MA: participated in surgery, reviewed the manuscript. CK: operating surgeon, aided in literature search. DW: aided in literature search. AH: aided in literature search. AK: operating surgeon, reviewed the manuscript. All authors read and approved the final manuscript.

## Authors’ information

CR: PhD candidate, Department of Cardiac Surgery, Vienna General Hospital, Medical University of Vienna.

MA: Resident, Department of Cardiac Surgery, Vienna General Hospital, Medical University of Vienna.

CK: Consultant, Department of Cardiac Surgery, Vienna General Hospital, Medical University of Vienna.

DW: Chief-resident, Department of Cardiac Surgery, Vienna General Hospital, Medical University of Vienna.

AH: PhD candidate, Department of Cardiac Surgery, Vienna General Hospital, Medical University of Vienna.

AK: Professor, Department of Cardiac Surgery, Vienna General Hospital, Medical University of Vienna.
